# Mitochondrial redox and pH signaling occurs in axonal and synaptic organelle clusters

**DOI:** 10.1038/srep23251

**Published:** 2016-03-22

**Authors:** Michael O. Breckwoldt, Antonis A. Armoundas, Miguel A. Aon, Martin Bendszus, Brian O’Rourke, Markus Schwarzländer, Tobias P. Dick, Felix T. Kurz

**Affiliations:** 1 Department of Neuroradiology, University of Heidelberg, Im Neuenheimer Feld 400, 69120 Heidelberg, Germany; 2 Cardiovascular Research Center, Harvard Medical School, Massachusetts General Hospital, Charlestown, MA, USA; 3 Division of Cardiology, Department of Medicine, Johns Hopkins University, Baltimore, MD, USA; 4 Laboratory of Cardiovascular Science, National Institute on Aging, Baltimore, MD, USA; 5 Institute of Crop Science and Resource Conservation (INRES), University of Bonn, Friedrich-Ebert-Allee 144, 53113 Bonn, Germany; 6 Division of Redox Regulation, German Cancer Research Center (DKFZ), DKFZ-ZMBH Alliance, Im Neuenheimer Feld 280, 69120 Heidelberg, Germany

## Abstract

Redox switches are important mediators in neoplastic, cardiovascular and neurological disorders. We recently identified spontaneous redox signals in neurons at the single mitochondrion level where transients of glutathione oxidation go along with shortening and re-elongation of the organelle. We now have developed advanced image and signal-processing methods to re-assess and extend previously obtained data. Here we analyze redox and pH signals of entire mitochondrial populations. In total, we quantified the effects of 628 redox and pH events in 1797 mitochondria from intercostal axons and neuromuscular synapses using optical sensors (mito-Grx1-roGFP2; mito-SypHer). We show that neuronal mitochondria can undergo multiple redox cycles exhibiting markedly different signal characteristics compared to single redox events. Redox and pH events occur more often in mitochondrial clusters (medium cluster size: 34.1 ± 4.8 μm^2^). Local clusters possess higher mitochondrial densities than the rest of the axon, suggesting morphological and functional inter-mitochondrial coupling. We find that cluster formation is redox sensitive and can be blocked by the antioxidant MitoQ. In a nerve crush paradigm, mitochondrial clusters form sequentially adjacent to the lesion site and oxidation spreads between mitochondria. Our methodology combines optical bioenergetics and advanced signal processing and allows quantitative assessment of entire mitochondrial populations.

Mitochondria play a crucial role in cellular energy supply, calcium buffering, ß-oxidation and homeostasis. Mitochondrial dysfunction has been implicated in a wide variety of diseases, including cardiovascular, neoplastic and neurological disorders^1^,^2^,^3^. It is now well established that mitochondria generate various forms of signals at the single organelle level: this includes redox signals that can regulate enzyme activity and transcription in part by modification of specific thiol residues^4^,^5^,^6^. Signal fluctuations have been described in single mitochondria and named “transients”, “pulses”, “oscillations”, “contractions” or “superoxide bursts”^7^,^8^,^9^,^10^,^11^. Although “superoxide bursts” are now recognized to actually represent “pH flashes”^12^, most of these signals are nevertheless associated with redox changes^7^,^8^,^9^,^10^,^13^ and implicated in disease or aging^8^,^14^,^15^.

Mitochondrial metabolism is determined by highly dynamic processes and small perturbations can result in collective mitochondrial behavior^16^,^17^. For instance, alterations in the cardiac redox environment can trigger mitochondrial inner membrane potential oscillations that scale from the level of single mitochondria to the level of the whole heart^18^. One emerging concept relates to inter-mitochondrial coupling^17^,^19^,^20^: mitochondria can function in synchronized functional units where they act collectively to allow metabolic fluxes and equilibrate the network^21^. A morphological coupling mode of adjacent mitochondria and the formation of inter-mitochondrial junctions has been recently described^22^. Mitochondrial signaling can also occur in a wave-like fashion across the mitochondrial network^8^,^23^,^24^,^25^,^26^. Such inter-mitochondrial coordination might be due to a dynamic equilibration of energetic states between neighboring mitochondria or the formation of signaling microdomains.

We have recently characterized a physiological redox signal in neuronal mitochondria that goes along with a profound shape change of the organelle (dubbed “mitochondrial contractions”)^8^,^27^. In this first description, mitochondrial redox signals were only investigated at a single organelle level. It remained unclear if such signals were sensed by adjacent mitochondria and if they could affect the mitochondrial population. Therefore, we now investigate the effect of mitochondrial oxidation and pH dynamics on the entire assessed mitochondrial population. By using optical sensors to measure the glutathione redox potential (E_GSH_) of neuronal mitochondria (in *Thy1*-mito-Grx1-roGFP2 mice) and mitochondrial matrix pH (in neurons transfected with AAV-mito-SypHer) we dissected mitochondrial redox and pH dynamics of a given axon (~25 mitochondria/axon) and neuromuscular junction (~50 mitochondria/NMJ) in parallel. We found that single organelle signals (glutathione oxidation/pH spikes) can occur multiple times within short time periods and are sensed by neighboring mitochondria to induce additional signals. Automated analysis allowed the precise extraction of various morphological and functional parameters (*e.g.* organelle size, location, relation to neighbors, signal rise time, amplitude, frequency) and their relation to the signal characteristics of the mitochondrial pool. We identified a higher order organization within morphologically and/or functionally coupled signaling clusters. The mitochondria-specific antioxidant MitoQ inhibited cluster formation whereas pathology (nerve crush) increased it. Our results reveal novel aspects of a collective behavior of neuronal mitochondria and provide further evidence of inter-organellar communication.

## Results

### A subpopulation of mitochondria exhibits multiple dynamic redox shifts

**Figure 1:**
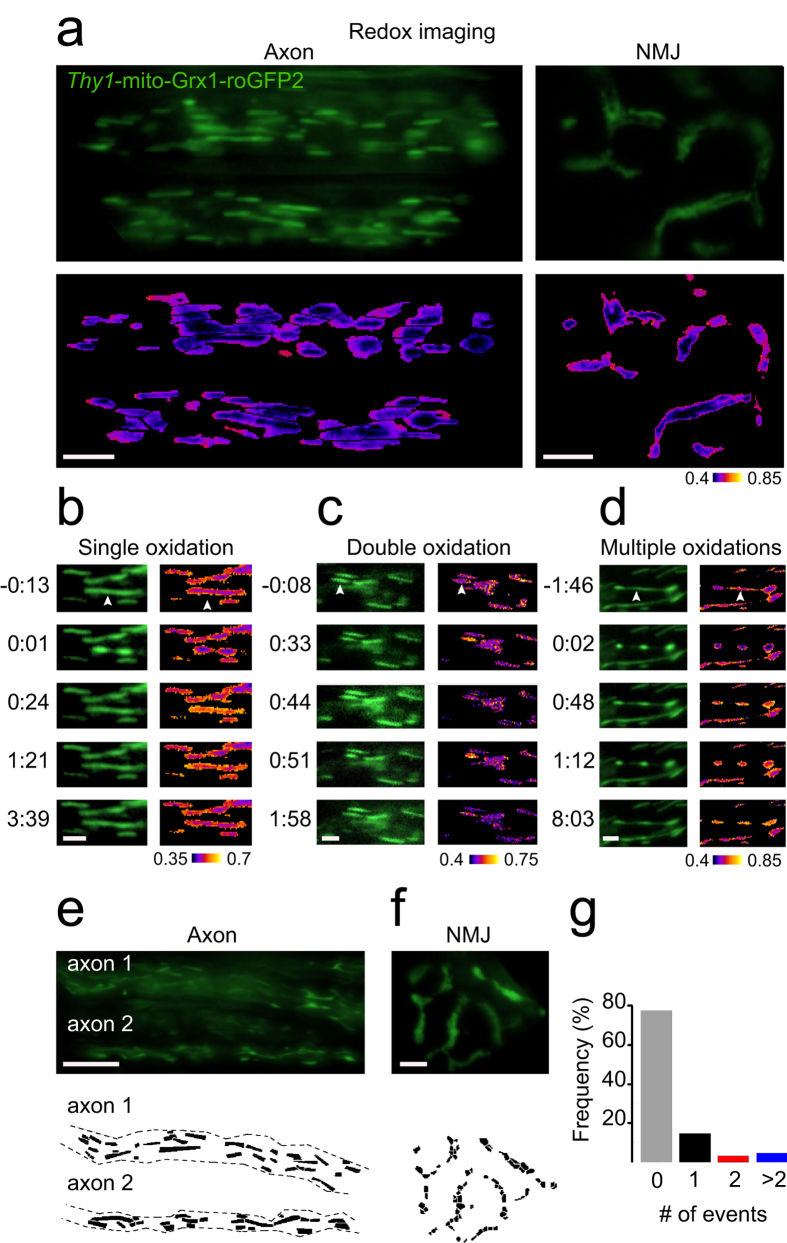
Illustration of redox imaging using grids. Redox imaging is performed in *Thy1*-mito-Grx1-roGFP2 triangularis sterni explants. Images show two axons (left) and a neuromuscular junction (NMJ, right). The mitochondrial probe oxidation is shown as pseudocolor ratio image of the 408/488 nm channel (below, **a**). Image illustrations of single, double and multiple oxidations of a single mitochondrion (**b–d**). Arrowheads indicate the contracting mitochondrion. Grids are used to read out the signal characteristics of all mitochondria of an imaging field within axons and the NMJ (**e,f**). Distribution of axonal E_GSH_ events (**g**). Scale bars in (**a**,**e**) (axon) = 10 μm; (**a,f**) (NMJ) = 5 μm; (**b**–**d**) = 2 μm.

The glutathione redox potential was assessed in single axonal and synaptic mitochondria of *Thy1*-mito-Grx1-roGFP2 mice expressing Grx1-roGFP2 in the mitochondrial matrix (Fig. 1a)^8^. Overall the majority of mitochondria showed stable E_GSH_ over the time of investigation (5–10 min/movie). However, we identified some mitochondria with markedly different redox characteristics: single, double or even multiple rounds of oxidation (>2 oxidation events and/or permanent oxidation) of the same organelle (Fig. 1b–d). Oxidation events were accompanied by morphological shortening and re-elongation cycles. Longer mitochondria exhibited a pearl-on-string morphology (Fig. 1b,d) whereas shorter mitochondria rounded up and subsequently re-elongated (Fig. 1c).

### Parallel assessment of redox and pH dynamics of entire mitochondrial populations

**Figure 2:**
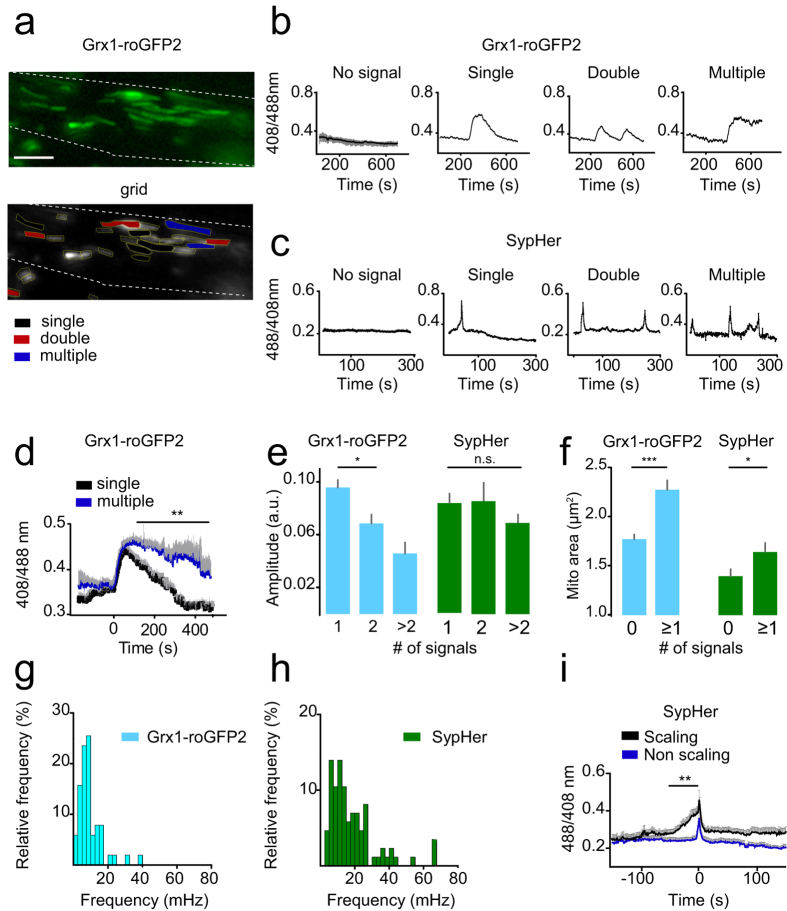
Parallel assessment of redox and pH dynamics of entire mitochondrial populations. Grid analysis allows readout of signal traces of single mitochondria exhibiting no signal changes, single, double and multiple/permanent oxidations in a representative axon (**a**). In the bottom image single event-mitochondria are shown with black overlay, double events with red overlay, multiple events with blue overlay and no events have no overlay. Mitochondrial matrix pH dynamics are assessed in AAV-mito-SypHer explants. Single, double and multiple pH spikes are found in individual mitochondria. Representative single traces are shown in (**c**). Single oxidation (black trace) compared to multiple oxidation traces (blue trace, **d**). *n* = 47 single oxidations and *n* = 35 multiple oxidations. Signal amplitudes in single and multiple event-mitochondria. E_GSH_ and pH amplitudes are shown (**e**). Mean mitochondrial area of event-mitochondria. Larger mitochondria are more likely to show redox or pH spikes (**f**). Differences in mitochondrial sizes might be due to the different techniques of fluorescent sensor expression (Grx1-roGFP2: transgenesis; SypHer: viral particles). Frequency of successive mitochondrial E_GSH_ and pH events (**g,h**). A subpopulation of mitochondria shows a scaling behavior of the SypHer signal (**i**). Scale bar in a = 5 μm. *p < 0.05, **p < 0.01, ***p < 0.001.

To quantify signal characteristics not only in single mitochondria but across the entire mitochondrial population, we developed advanced automated quantification algorithms. We customized a previously developed technique using image grids and advanced Matlab routines^10^,^28^. This enabled us to characterize the signal dynamics of large mitochondrial populations in axons and synapses over time. Up to ~100 mitochondria (~25 mitochondria/axon; ~50 mitochondria/synapse) were covered simultaneously in 5–12 min long movies (imaging speed: 1 Hz) leading to ~72.000 individual data points (Fig. 1e,f). We characterized the signal traces of >1500 mitochondria. Overall, single axonal redox events occurred in ~18% of mitochondria. The automated analysis greatly aided the identification of sparser events which occurred in ~8% of mitochondria (double and multiple oxidations: *n* = 67 in a total of *n* = 817 Grx1-roGFP2 mitochondria; Fig. 1g, Suppl. Table 1). Redox traces could be divided into “no activity”, “single oxidation”, “double oxidation” or “multiple oxidation events” (Fig. 2a,b, Suppl. Figs 1 and 2). To gain insight into the bioenergetic properties of mitochondria during the oxidation events, we injected AAV1/2-mito-SypHer^11^ into spinal motorneurons of wild type mice and measured the matrix pH dynamics, as an indicator for the proton gradient (ΔpH) across the inner membrane (Suppl. Fig. 3). ΔpH and electrical potential ΔΨ constitute, the mitochondrial proton motive force (PMF). Together, redox and pH measurements give an estimate of mitochondrial bioenergetics. Similar patterns of activity with single, double and multiple pH flashes within single mitochondria could be discerned (Fig. 2c).

Further analysis showed that multiple oxidation events led to a persistent elevation of E_GSH_ over at least ~5 min whereas E_GSH_ returned to baseline within ~200 seconds after single oxidation events (Fig. 2d). Oxidation amplitudes became smaller after multiple rounds of contraction-relaxation cycles, whereas pH amplitudes stayed constant (Fig. 2e, Suppl. Table 2). This indicates that the glutathione pool oxidizes progressively following successive events whereas the matrix pH re-equilibrates faster.

### Automated morphological and signal analysis can link mitochondrial morphology and function

Grid analysis allocates a unique identifier to each mitochondrion that allows linking morphological and functional information. We assessed mitochondrial length, width and area, as well as redox and pH event frequency, amplitude, rise and decay time and slope (Suppl. Fig. 4, Suppl. Table 2). Detailed morphological characteristics are shown in Suppl. Fig. 4c,d. Of note is that mitochondria within the axon were larger compared to synaptic mitochondria and showed higher signal amplitudes than synaptic mitochondria (Suppl. Fig. 4d,e). Interestingly, the baseline E_GSH_ and matrix pH were higher in mitochondria displaying multiple contractions as compared to non-contracting mitochondria (Grx1-roGFP2: 408/488 nm: 0.38 ± 0.01 *vs.* 0.30 ± 0.02, p < 0.01, SypHer: 488/408 nm: 0.31 ± 0.01 *vs.* 0.25 ± 0.01, p < 0.05, Suppl. Fig. 5). This indicates that event-mitochondria show a shift in their bioenergetic steady-state. Mitochondria that showed events were overall larger than silent mitochondria (mitochondrial area: 2.27 ± 0.10 μm^2^ *vs.* 1.77 ± 0.05 μm^2^, p < 0.001, Fig. 2f). Also, the frequency of pH transients was higher compared to E_GSH_ events (Grx1-roGFP2: 1.02 ± 0.09 × 10^−2^ Hz, *n* = 52 mitochondria with multiple events *vs.* SypHer: 1.84 ± 0.15 × 10^−2^ Hz, *n* = 86, p < 0.001, Fig. 2g,h). This may be interpreted as a more dynamic behavior of pH changes (proton flux) than redox changes. Yet, SypHer reacts faster than Grx1-roGFP2 and with particularly high sensitivity as it only depends on direct protonation/de-protonation of the chromophore, as opposed to the multi-step thiol-disulfide exchange mechanism of Grx1-roGFP2, and has a larger spectroscopic dynamic range than Grx1-roGFP2. These differences in sensor properties are likely to explain differences in sensitivity and therefore a strict linkage of pH and E_GSH_ transients remains likely.

### Characterization of individual events

The complexity of mitochondrial networks is typically associated with changing characteristics that are not necessarily stationary in time^10^,^29^. Wavelet transforms are a powerful method to decipher the time-dependency of the dominating frequencies of a signal and, thus, can be helpful to better characterize individual Grx1-roGFP2 and SypHer signals (Suppl. Fig. 6a,b)^30^. For Grx1-roGFP2 traces, wavelet transforms did not reveal additional frequency bands besides frequencies of multiple, subsequent events (red area in the wavelet representation in Suppl. Fig. 6a). The characteristic sharp events in the SypHer traces, however, were often associated with smeared high-frequency areas in their wavelet representations and a sharper dynamic frequency trace of subsequent events (Suppl. Fig. 6b). As higher frequencies (up to ~200 mHz) also occurred in the vicinity of some SypHer peaks we examined whether a slow matrix alkalization precedes the pH flash.

### Mitochondrial signal scaling

While most SypHer traces showed a stereotypical signal with a sudden pH rise (alkalization, Fig. 2c), some events were preceded by a characteristic slow baseline ascent or a small wave-like signal (Fig. 2c right panel, Fig. 2i, Suppl. Fig. 7a). This pre-signal could be attributed to a scaling phenomenon where a slow pH rise leads to a sudden over-activation of the electron transport chain and the fast extrusion of protons from the matrix that constitutes the actual pH spike. A signal scaling was not apparent for Grx1-roGFP2. Within the time-to-peak times for all SypHer events (Suppl. Fig. 7b), a small peak was present at ~25–30 s as opposed to the main distribution peak at ~10 s. The ascent time for a scaling event was 30.08 ± 2.48 s with a mean slope of 0.42 ± 0.08 × 10^−2^ s^−1^ (*n* = 12 traces). We sought to determine the percentage of such scaling traces and included traces with a time to peak time of 30.08 s ± one standard deviation (SD). According to this definition, ~17% of all SypHer events showed a scaling character. We assume that such scaling behavior can either be attributed to the local micro-environment in the surrounding cytoplasm or to an intrinsic mitochondrial phenomenon.

### Mitochondrial events occur in local clusters

**Figure 3:**
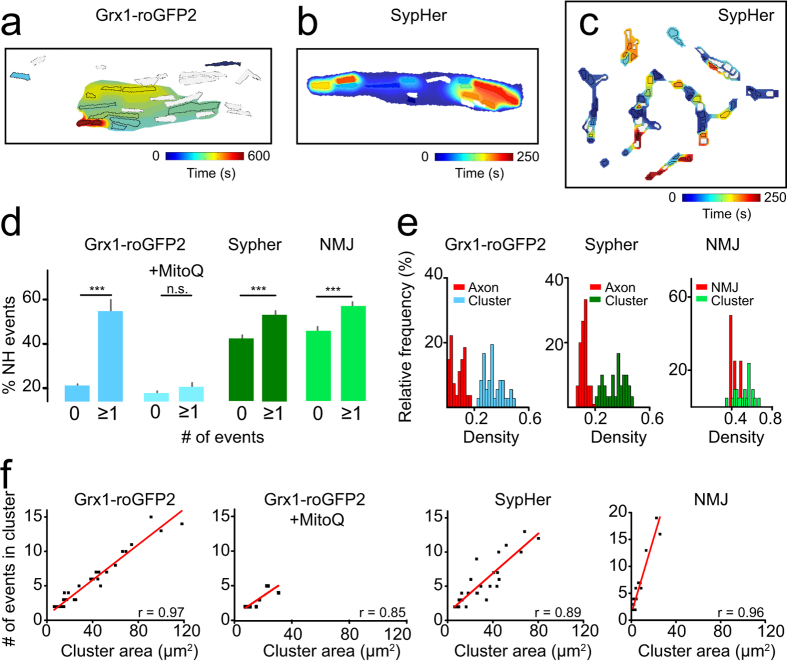
Mitochondrial signals occur in signaling clusters. Isochrone analysis of E_GSH_ and pH events within axons (**a,b**) and at the neuromuscular junction (**c**). White mitochondria show no event. Signal events are clustered and subsequent events originate more likely in the neighborhood (NH) of event-mitochondria (**d**). The antioxidant MitoQ (1 μM) blocks cluster formation. Grx1-roGFP2, MitoQ and SypHer were recorded in the axon. Comparison of mitochondrial densities within clusters and the entire axon, and comparison of mitochondrial and cluster densities in the NMJ (**e**). Correlation analysis of cluster size and event frequency (**f**). ***p < 0.001.

Multiple events of the same organelle may indicate that local factors trigger the contraction process. Such factors could be envisioned to act more broadly within the local mitochondrial population. To analyze a potential environmental effect, and whether signaling occurs between mitochondria, we first examined the spatial proximity of mitochondria that exhibited events in axons and at the neuromuscular junction. We found strong indications that pro-oxidative E_GSH_ shifts and pH spikes indeed occur in organelle clusters (Fig. 3a–c, Suppl. Fig. 8, see Methods for cluster definition). Isochrone analysis was used to evaluate spatial and temporal clustering of events.

Among all events within a recording, the earliest mitochondrial event was chosen as a reference time-point and the difference of all following events was determined to create isochronal maps (Fig. 3a–c). Isochronal maps demonstrate the close spatial and temporal relationship of events. In Fig. 3a (Grx1-roGFP2), the earliest events arise from spatially clustered mitochondria in the bottom center of the axon and subsequent events in neighboring mitochondria follow with a delay of ~100 s, suggesting a spread velocity of ~40 μm/s. In Fig. 3b (SypHer), mitochondria at both ends of the axon, show an event at ~200–250 s after the earliest event. Assuming uniform spread of the signal from the first mitochondrion to the farthest ends of the axon, the spread velocity corresponds to ~45–55 μm/s. Event clusters at the neuromuscular junction can also frequently be discerned (Fig. 3c). Other axons exhibited only small clusters or did not demonstrate obvious local clustering of events (Suppl. Fig. 9). This might be explained by the fact that the activity of mitochondrial signals within the axon (or neuromuscular junction) is normally distributed with local hotspots and less active regions.

To further analyze more quantitatively whether event clustering occurs, we calculated the probability of finding neighborhood (NH) events in the vicinity of event-exhibiting mitochondria. Indeed, we found a significantly higher probability of additional signals in the respective neighborhood of signaling mitochondria (Fig. 3d). This was the case for axonal redox and pH signals as well as synaptic signals (p < 0.001, Fig. 3d). Cluster activity was redox dependent and was mitigated by antioxidant treatment with MitoQ (1 μM, Fig. 3d). Mitochondrial density within clusters was 10-fold higher as compared to the overall mitochondrial density within the axon (mitochondrial density in clusters_Grx1-roGFP2_ 0.34 ± 0.07, *n* = 36 clusters *vs.* axon_Grx1-roGFP2_ 0.03 ± 0.02, *n* = 27 axons, p < 0.001, Fig. 3e). Mitochondrial density impacts the event-probability of mitochondria: synaptic clusters were more numerous than axonal clusters (number of clusters: NMJ 5.33 ± 0.88 *vs.* axon 1.57 ± 0.17, p < 0.01) and had higher mitochondrial and event densities (mitochondrial density: clusters_NMJ_: 0.47 ± 0.02, *n* = 21, entire NMJ: 0.43 ± 0.02, *n* = 4 Fig. 3e; event density: axonal clusters (SypHer) 0.15 ± 0.01 μm^–2^; NMJ clusters 0.70 ± 0.05 μm^–2^ Fig. 3f).

### Morphological basis for local clustering

**Figure 4:**
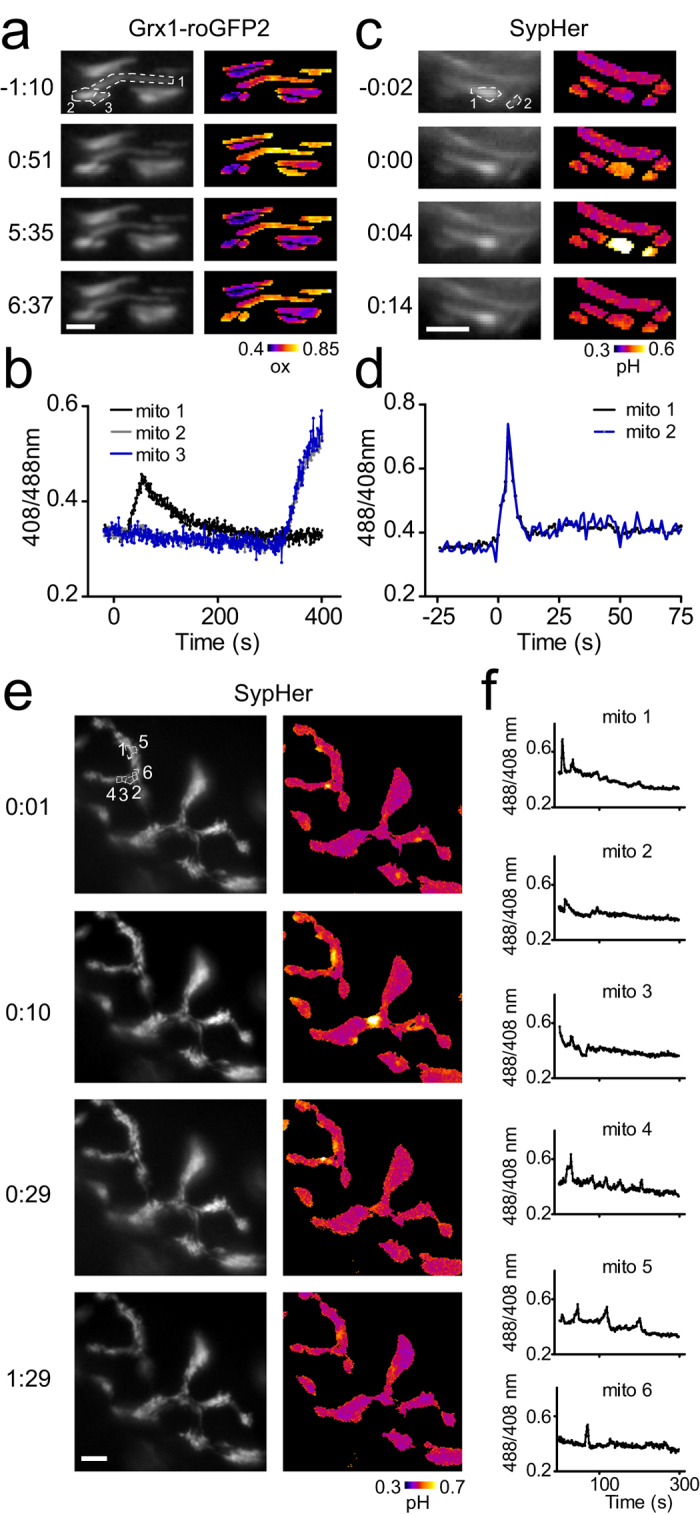
Mitochondrial signals influence surrounding mitochondria. Two adjacent mitochondria within a highly active cluster (5/8 mitochondria oxidize within the imaging period) oxidize simultaneously exhibiting an almost identical trace (mitochondrion 2 and 3; **a,b**). pH alterations can spread between mitochondria (**c,d**). Clustered pH signals at the NMJ (**e,f**). Scale bars in a and c = 2 μm, e = 5 μm.

Next we sought to better understand the basis for the observed spatial and temporal signal accumulation. Interestingly, we often found close morphological proximity between mitochondria undergoing events. In these instances, immediate proximity between neighboring mitochondria may involve structural contact, even though shared ultrastructures like intermitochondrial junctions could not be directly resolved by conventional light microscopy. In such neighboring mitochondria, an instantaneous spread of either glutathione oxidation or the pH signal was observed (Fig. 4a–d). Synaptic clusters could also be frequently detected and occurred in densely-packed areas (Fig. 4e,f). Notably, such signals were not strictly time-locked, arguing that local factors might built up within signaling hotspots.

### Clustering under pathological conditions

**Figure 5:**
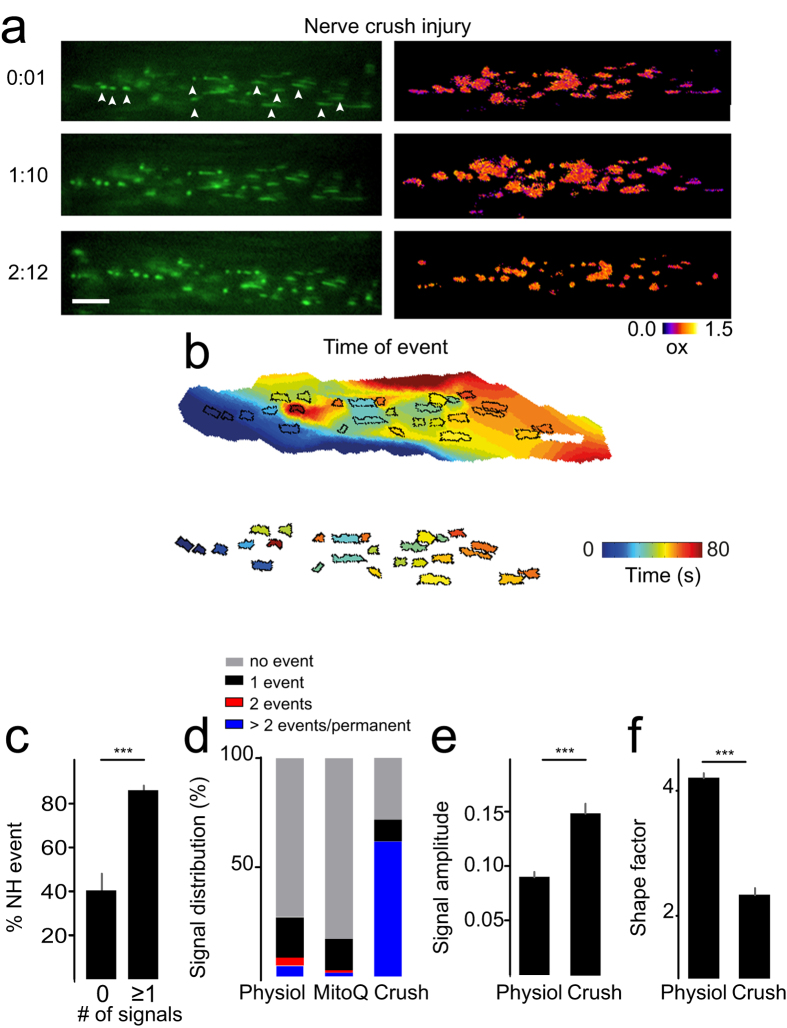
Mitochondrial signals after nerve crush injury. Illustration of E_GSH_ in a mito-Grx1-roGFP2 triangularis sterni explant after crush injury. Mitochondrial rounding and oxidation occurs subsequently after the crush from proximal (left) to distal (right) of the crush (**a**). Arrowheads indicate a selection of event-mitochondria. Isochrone analysis shows spreading and clustered oxidation. White mitochondrion shows no event (**b**). Neighborhood events are more likely to occur next to event-mitochondria (**c**). Signal distribution of mitochondria under physiological, MitoQ (1 μM) and crush conditions (**d**). Signal amplitudes are increased (**e**) and mitochondrial shape factor is decreased after the crush (**f**). Scale bar in a = 5 μm. ***p < 0.001.

To determine the effect of acute neuronal injury on cluster formation, we performed a mechanical nerve crush injury in triangularis sterni explants. Axonal crush led to permanent mitochondrial rounding which spread away from the crush site to more distally located mitochondria (Fig. 5a). Spatial and temporal clustering occurred even more frequently than under physiological conditions (Fig. 5b,c). Overall, ~61% of mitochondria showed permanent rounding along with a distinct Grx1-roGFP2 signal indicating persistent glutathione oxidation which had a higher amplitude compared to physiological E_GSH_ signals (amplitude: physiological event 0.09^ ^±^ ^0.01 *vs.* nerve crush 0.15^ ^±^ ^0.01, p < 0.001, Fig. 5d–f, Suppl. Table 1).

## Discussion

Mitochondrial transients have emerged as an intriguing topic in cell biology. They were described in different forms as “spontaneous” fluctuations of ΔΨ, matrix pH, ROS or E_GSH_ and found in diverse systems such as yeast, Arabidopsis root cells, cell cultures, cardiomyocytes and neurons^7^,^8^,^9^,^10^,^11^. There are several studies that observed or analyzed mitochondrial network characteristics, signal synchronization, inter-mitochondrial coupling and functional connectedness in yeast, salivary glands, cardiac cells and neurons^17^,^22^,^31^,^32^,^33^. In the present study, we provide an integrative network perspective by assessing the effect of individual mitochondrial signals on surrounding mitochondria and the entire mitochondrial population of a given imaging field. This was possible through the parallel and automated readout of all signal traces of the imaged mitochondria using grid templates with advanced image and signal processing routines. Thus, we could analyze the E_GSH_ and pH dynamics of up to ~100 mitochondria simultaneously. This allowed us to describe more infrequent events (like double or multiple oxidations) as well as the effect of single events on surrounding mitochondria.

In many instances, transients did not occur randomly but rather within spatially and temporally linked event clusters. Whether mitochondrial events are spatio-temporally clustered or occur independently of neighboring mitochondria is most likely linked to differences in mitochondrial morphology and function. Mitochondrial functional heterogeneity has been attributed to differences in respiratory chain activity, calcium, ‘ROS’, membrane potential and redox states^34^. The observation of mitochondrial subsets with a specific subcellular location (*e.g.* perinuclear or synaptic as in cardiac cells or neurons, respectively) or with specific surroundings (*e.g.* embedded in a collection of event-mitochondria; proximity to other organelles like the endoplasmatic reticulum) suggest an additional layer of complexity. The present study supports the concept of morphological and functional heterogeneity: mitochondria undergoing transients are significantly larger than silent mitochondria (Fig. 2f). Also, it is more likely to find signaling mitochondria in the local neighborhood of events (Fig. 3d). Axonal mitochondria showed higher signal amplitudes (Suppl. Fig. 4e); as axonal mitochondria are larger than synaptic mitochondria they possess larger membrane surfaces they might also exhibit more contact sites to other mitochondria. In the same line of reasoning we observed that mitochondrial density within clusters was higher than within the rest of the axon (Fig. 3e). It is therefore likely that such events involve ionic exchange and possibly other substances for equilibration of the mitochondrial network.

Furthermore, we observed a strong local clustering of pH signals, especially within synapses (Fig. 4e,f), suggestive of local signaling microdomains^35^. In contrast to axonal mitochondria, the investigation of synaptic mitochondria possess a challenge for light microscopy due to higher mitochondrial densities and overall smaller sizes of NMJ mitochondria. In our analysis, the mean mitochondrial area within the synapse was 0.52 ± 0.26 μm^2^ (Suppl. Fig. 4d) which is in line with electron microscopy studies (mitochondrial area: ~0.1–2.2 μm^2^)^36^,^37^. The limited resolution of conventional light microscopy results in uncertainty for the selection of grids, especially when mitochondria are in immediate contact. Even though the grid selection of a given NMJ mitochondria might be arbitrary in some instances, we are confident that this is not a major bias for our results: our mean cluster size in synapses was 5.88 ± 1.46 μm^2^. This is much larger than a single mitochondrion, indicating that the sampled signals (6.25 ± 1.31 events/cluster) are derived from distinct mitochondria.

Mechanistically, event clustering was suppressed by the antioxidant MitoQ, suggesting a redox dependent coupling mechanism. Mitochondrial events after MitoQ application were generally sparse (Fig. 5d) and clusters remained small (Fig. 3f). Under pathological conditions, nerve crush injury led to mitochondrial rounding (Fig. 5f) and an increase of high amplitude mitochondrial signals compared to physiological conditions (Fig. 5d,e). Hence, mitochondrial contractions and accompanying redox/pH fluctuations may represent a physiological stress response that is aggravated under pathological conditions and can become irreversible, thus leading to permanent mitochondrial damage and neuronal demise.

A spatio-temporal organization of mitochondria into clusters has so far mainly been described in cell culture^38^,^39^ and intact perfused organs^20^, but has not been quantitatively described in neurons. Quantitative network analyses based on individual mitochondrial signals have only recently been applied to mitochondrial lattice-like networks^10^,^20^. Prior studies were performed in mitochondrial networks with reticular organization^40^ or using biochemical models^41^. While ROS-induced ROS release was proposed as an inter-mitochondrial coupling mechanism in cardiac cells^42^, direct morphological inter-mitochondrial coupling in neurons might be based upon various recently described phenomena such as mitochondrial kissing or nano-tunneling^43^. Intermitochondrial junctions in cardiac cells showed shared cristae between adjacent mitochondria^22^. Our study is in line with the idea of partial membrane fusion that might occur between mitochondria during shared events^22^,^38^, even though we were not able to demonstrate this directly due to the limited resolution of conventional light microscopy that we used. Electron microscopy could resolve such contact sites. In our paradigm, a correlated light and electron microscopy approach however would be highly challenging as it would require to first functionally map the inherent transient mitochondrial signal by light microscopy and then re-identify the same mitochondrion on serial section EM to resolve the entire axon/NMJ.

Moreover, mitochondria without evident structural contact still exhibit signaling-mediated collective behavior within localized clusters. We found that signal propagation can occur between individual mitochondria at a velocity of 40–55 μm/s. Such a velocity is in the range of other studies examining the propagation of mitochondrial inner membrane depolarization for lattice-organized mitochondria in cardiac myocytes (22 and 32 μm/s, respectively^10^,^25^).

One limitation of our study concerns the fluorescent proteins employed. As Grx1-roGFP2 and SypHer are both excited ratiometrically at 408/488 nm we could not co-image the sensors in the same preparation. However, recent work strongly suggests that both signals are closely linked and that redox and pH signals coincide^7^,^8^,^38^.

In summary, using novel multi-parametric imaging techniques, we show that individual mitochondria within neurons can exhibit collective behavior, appear to signal within local clusters and respond to the activity of other mitochondria. Our analysis sheds light on inter-mitochondrial communication within neurons under physiological and pathological conditions. As such it provides an important step towards deciphering mitochondrial signaling. Future research should further elaborate on the role and functional implications of mitochondria-dependent axonal and synaptic signaling and how these signals are transduced to other subcellular compartments (*e.g.* nucleus, ER). Altogether the mitochondrial transients reported for several physiological parameters and in different systems (“transient”, “pulse”, “pH flash”, “oscillation” or “contraction”) may be interpreted as a mitochondrial signaling language that is now being deciphered and that is likely related and conserved from plants to mammals.

## Material and Methods

### Experimental methods

#### Animals

Generation of transgenic *Thy1*-mito-Grx1-roGFP2 (roGFP2) mice has been described previously^8^. We used adult male and female mice (older than 6 weeks and up to ~8 months of age) for our experiments. All animal work conformed to institutional guidelines and was approved by the Animal Study Committee of the Regierung von Oberbayern.

#### Preparation of triangularis sterni explants

Triangularis sterni explants were prepared as previously described^44^,^45^. Briefly, mice were euthanized with isoflurane, and the rib cage (with the attached triangularis sterni muscle and its innervating intercostal nerves) was isolated by paravertebral cuts, pinned in a sylgard-coated dish using insect pins and maintained on a heated stage (32–35 °C) in normal Ringer’s solution containing (in mM) (125 NaCl, 2.5 KCl, 1.25 NaH_2_PO_4_, 26 NaHCO_3_, 2 CaCl_2_, 1 MgCl_2_ and 20 glucose), bubbled with 95% O_2_, 5% CO_2_. Imaging was performed up to ~4 hours after dissection to ascertain physiological conditions. For the nerve crush experiment mechanical pressure was applied with small forceps under visual guidance for 5–10 s on the intercostal nerve. Imaging started immediately after the crush adjacent to the lesion site. Pharmacological experiments were performed with MitoQ (1 μM, kind gift of M. Murphy, University of Cambridge).

#### pH measurements using AAV-mito-SypHer

To assess the mitochondrial matrix pH in triangularis sterni explants, we injected recombinant adeno-associated virus particles (rAAV-1/2-mito-SypHer, containing a 1:1 ratio of AAV1 and AAV2 capsid proteins) into the cervical spinal cord, targeting motor neurons in the ventral horn that give rise to intercostal axons that project to the triangularis sterni muscle. The injection level was C7-T1, one mm lateral to the midline at a depth of 0.9 mm below the dural surface. Each virus was diluted 1:2 in sterile phosphate buffered saline (PBS). rAAV-mito-SypHer (Sypher; matrix pH, titer 1.01 × 10^11^ particles/ml) was cloned and produced in AAV-293 (Invitrogen) by standard methods^46^,^47^. Triangularis sterni explants of black 6 WT mice injected with rAAV-1/2-mito-SypHer were prepared 10–14 days after virus injection and performed as described above. SypHer recordings at the NMJ were performed with 100 μM genipin (Sigma) added to Ringer’s solution.

#### Image recording and microscopy

To record mitochondrial redox and pH dynamics in motor axons and NMJs, we used a BX51 wide-field microscope (Olympus) equipped with ×20/0.5 NA and ×100/1.0 NA dipping-cone water-immersion objectives, a cooled charged-coupled device camera and a PolyV polychromator system (Till Photonics, controlled by TillVision software; dichroic filter: D/F 500 DCXR; emission filter: ET 525/36). Images were acquired at rates of 1 Hz with exposure times of 150 (450) ms for 408 nm excitation and 30 (150) ms for 488 nm excitation. Some of the axon movies have been previously manually analyzed and published in the context of single contractions^8^.

### Extraction and analysis of individual mitochondrial signals

#### Image post-processing

The translational drift in x-/y-direction for subsequent images in each recording was corrected with the image stabilizer plugin for ImageJ (Version1.49s)^48^. For each recording, the background signal (outside of the axon/NMJ) was subtracted in both channels.

#### Selection of individual mitochondrial fluorescence traces

For each recorded movie, the average projection was calculated and uploaded into Adobe Photoshop CS6 v13.0. The contour of every individual mitochondrion in the average image was manually drawn on a pixel-by-pixel basis. Furthermore, axon borders were drawn and the space in between mitochondrial contours and within axon borders was assigned as cytoplasm. In this ternary grid template, every mitochondrion received a numerical identifier. Individual mitochondrial intensity traces for both the 408 nm and the 488 nm channel were obtained as the average pixel intensity at each time-point for the pixels within and including the respective mitochondrial contour. For Grx1-roGFP2, the mitochondrial intensity trace was determined as the ratio of the signal of the 408 nm and the 488 nm channel, whereas SypHer traces were determined as the ratio of the 488 nm and the 408 nm channel.

#### Peak selection and signal characteristics

Events for a mitochondrial fluorescence trace were defined as every deviation of more than 10% relative to the intensity trace baseline, and were determined using custom-written Matlab code. Thereby, event onset (*t*_0_), end (*t*_end_) and peak were identified and time to peak (Δ*t*_up_) and time to decay (Δ*t*_down_) were determined (Suppl. Fig. 1). In addition, the amplitude (ΔA) was obtained as the absolute fluorescence ratio difference between the event onset and Δ*t*_up_ after onset. To determine the rise of the redox or pH event, only time-points in the interval (*t*_0_ + 0.1 · Δ*t*_up_, *t*_0_ + 0.9 · Δ*t*_up_) were considered: for these time-points, the intensity trace was fitted with a linear polynomial to determine the linear slope *c* (Suppl. Fig. 1). The characteristic decay of the event was fitted with the exponential function *s*(*t*) = *s*_0_ + *b*∙exp(−*a*∙*t*) to determine the decay slope *a*.

#### Mitochondrial event frequencies

In case of multiple events in a mitochondrial trace, the frequency of subsequent events was determined as the inverse of the difference of subsequent peak time-points.

#### Wavelets analysis

The wavelet transform provides a frequency-time representation that shows the hierarchy of dominant frequencies for every time-point of a time series in color code (Suppl. Fig. 6). To demonstrate high-frequency content in SypHer fluorescence traces (see Suppl. Figs 6b and 7), the Morlet wavelet with its higher frequency resolution was preferred over other wavelet forms. The smallest wavelet scale *s*_0_ was set as the smallest scale to detect one oscillatory cycle *s*_0_ = 4d*t*, where d*t* represents the sampling rate and the spacing between different wavelet scales was set at d*j* = 0.1. The number of scales was fixed as *j*_1_ = log_2_(*T*/*s*_0_)/d*j* + 1 (*T* being the total number of recorded images). This leads to scales ranging from *s*_0_ to *s*_0_2^(j1-1)∙dj^ and each scale with d*j* suboctaves. The upper cutoff frequency was chosen as the inverse of 1.1∙(Δ*t*_up_ + Δ*t*_down_) and the lower cutoff frequency as 1/*s*_0_.

### Mitochondrial morphology, local neighborhoods and event clusters

#### Mitochondrial morphological characteristics

Each mitochondrial area was determined from the amount of pixels of the respective mesh within the grid template. Furthermore, the mitochondrial shape factor was obtained as the ratio of mitochondrial major and minor axis length.

#### Local mitochondrial neighborhoods

For each mitochondrion, local neighborhoods (NH) were determined as the surrounding area of cytoplasm within a radius of 4 μm around its center-point (corresponding to twice the mean length of one mitochondrion, *cf.* Suppl. Fig. 4c,d).

#### Mitochondrial clusters

Assemblies of mitochondria that exhibited events were determined from each recording as the area spanned by these mitochondria after performing morphological closing with a disk of 4 μm diameter. Morphological closing on a binary image consists of a dilation and erosion with a structuring element (*e.g.* disk) to obtain areas of clustered elements. This procedure yielded either isolated mitochondria or clusters of more than one mitochondrion (Suppl. Fig. 8). Cluster densities were determined as the ratio of the sum of mitochondrial areas within the cluster area to the cluster area. To compare these densities with axon densities, only the axon area spanned by all mitochondria within the axon was considered and the axon density was determined as the ratio of the sum of mitochondrial areas to the axon area.

### Statistical analysis

Image processing algorithms, fitting routines and the wavelet analysis were performed using Matlab v7.14.0.0739 (R2012a). Statistical analysis was performed in OriginPro 8 SR0 v8.0724 (B724) or PRISM (Graphpad). Data is presented as mean and standard error of the mean (SEM), unless stated otherwise. Statistical significance was determined with two-tailed t-tests or analyses of variance (ANOVA) with post hoc Fisher tests in cases of multiple comparisons. *indicates p < 0.05, **p < 0.01, ***p < 0.001.

## Additional Information

**How to cite this article**: Breckwoldt, M. O. *et al*. Mitochondrial redox and pH signaling occurs in axonal and synaptic organelle clusters. *Sci. Rep.* **6**, 23251; doi: 10.1038/srep23251 (2016).

## Supplementary Material

Supplementary Information
