# Supplementary material for: Mitochondrial redox and pH signaling occurs in axonal and synaptic organelle clusters
Source: Sci Rep. 2016 Mar 22;6:23251. doi: 10.1038/srep23251 (PMC4802380; doi:10.1038/srep23251)
Supplement: Supplementary Information [file srep23251-s1.pdf]

# - *Supplementary information* -

Mitochondrial redox and pH signaling occurs in axonal and synaptic organelle clusters

Michael O. Breckwoldt[1,*], Antonis A. Armoundas[2,+], Miguel A. Aon[3,4,+], Martin Bendszus[1], Brian O`Rourke[3], Markus Schwarzländer[5], Tobias P. Dick[6], Felix T. Kurz[1,2,*]

[1]Department of Neuroradiology, University of Heidelberg, Im Neuenheimer Feld 400, 69120 Heidelberg, Germany
[2]Cardiovascular Research Center, Harvard Medical School, Massachusetts General Hospital, Charlestown, MA, USA
[3]Division of Cardiology, Department of Medicine, Johns Hopkins University, Baltimore, MD, USA
[4]Laboratory of Cardiovascular Science, National Institute on Aging, Baltimore, MD, USA
[5]Institute of Crop Science and Resource Conservation (INRES), University of Bonn, Friedrich-Ebert-Allee 144, 53113 Bonn, Germany
[6]Division of Redox Regulation, German Cancer Research Center (DKFZ), DKFZ-ZMBH Alliance, Im Neuenheimer Feld 280, 69120 Heidelberg, Germany

Suppl. Fig.1

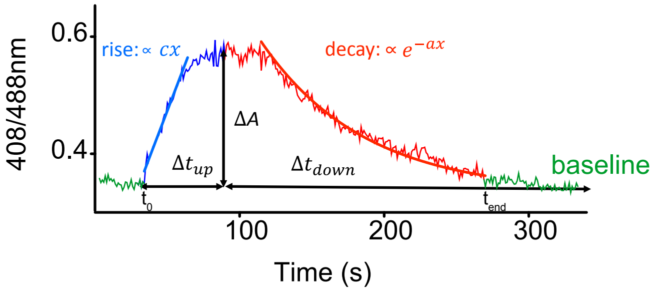

Suppl. Fig.2

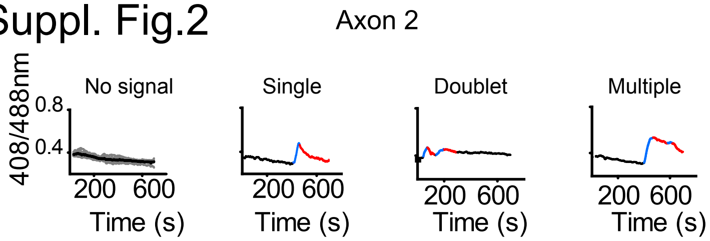

Suppl. Fig.3

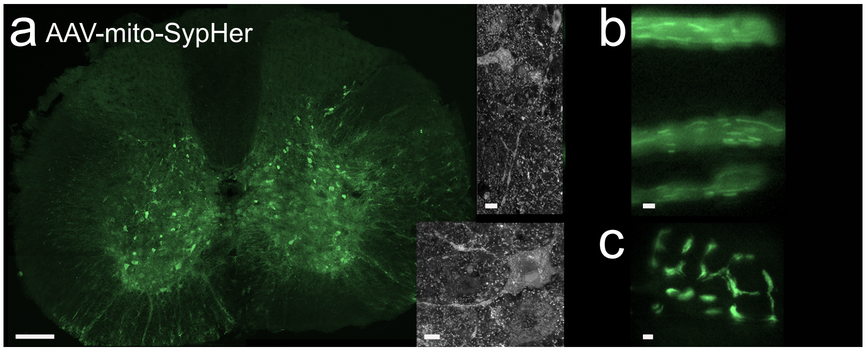

Suppl. Fig.4

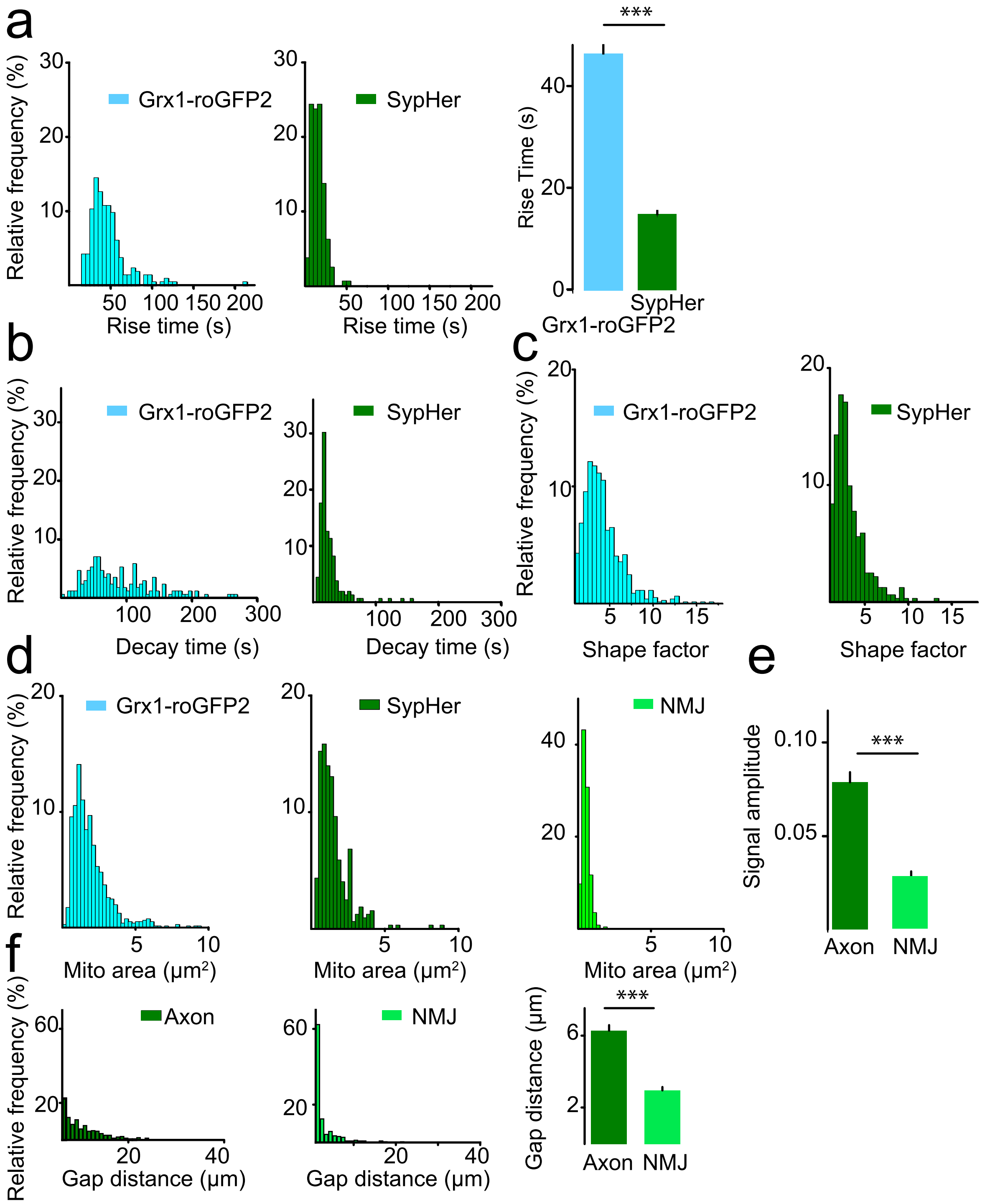

Suppl. Fig.5

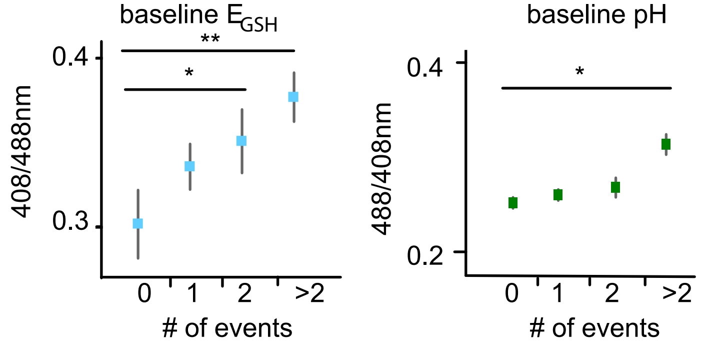

Suppl. Fig.6

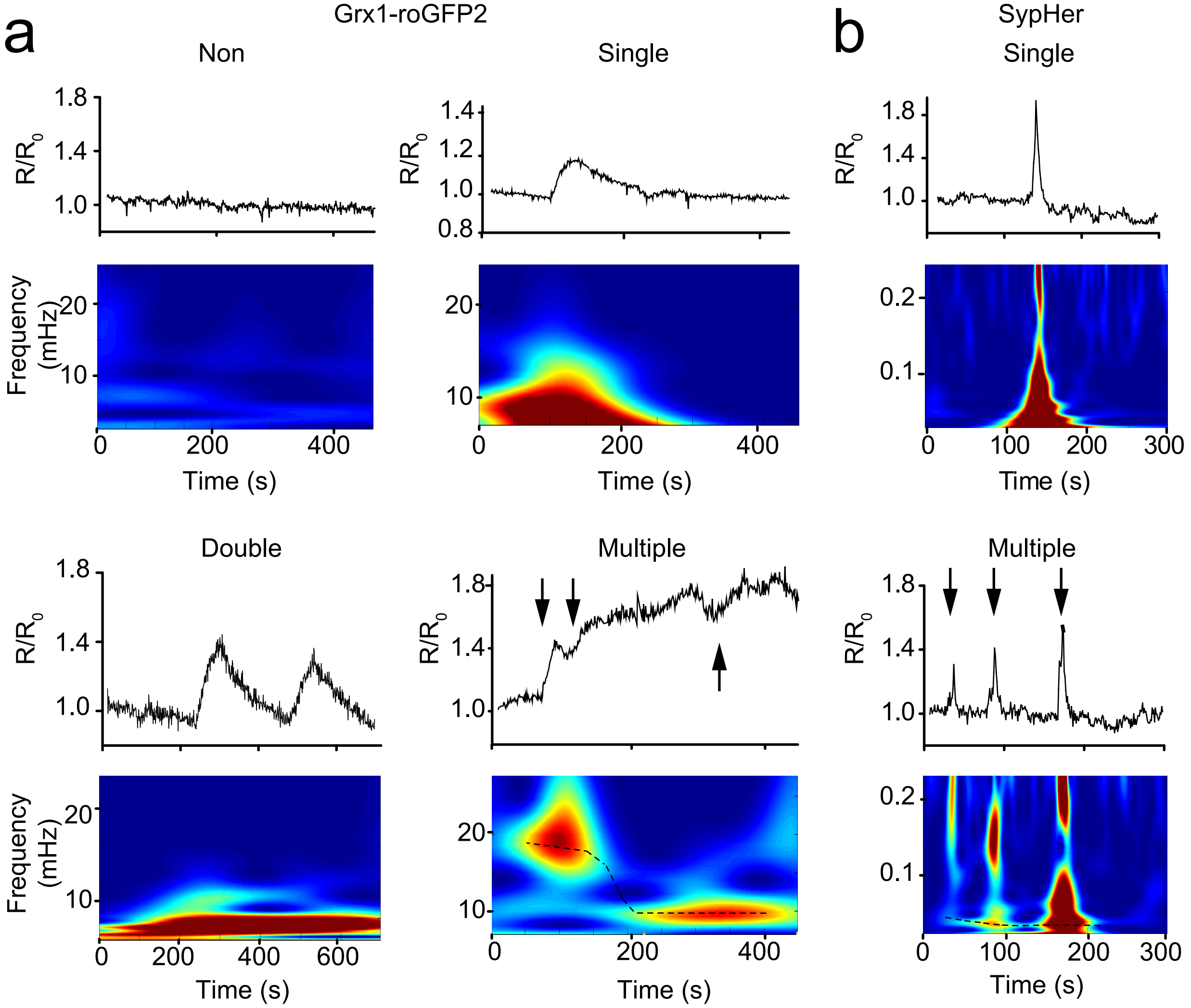

a
Grx1-roGFP2
Non
Single
b
SypHer
Single
R/R0
1.8
1.4
1.0
1.2
0.8
0.2
0.1
Frequency (mHz)
20
10
0
200
400
100
300
Time (s)
Double
Multiple
600

Suppl. Fig.7

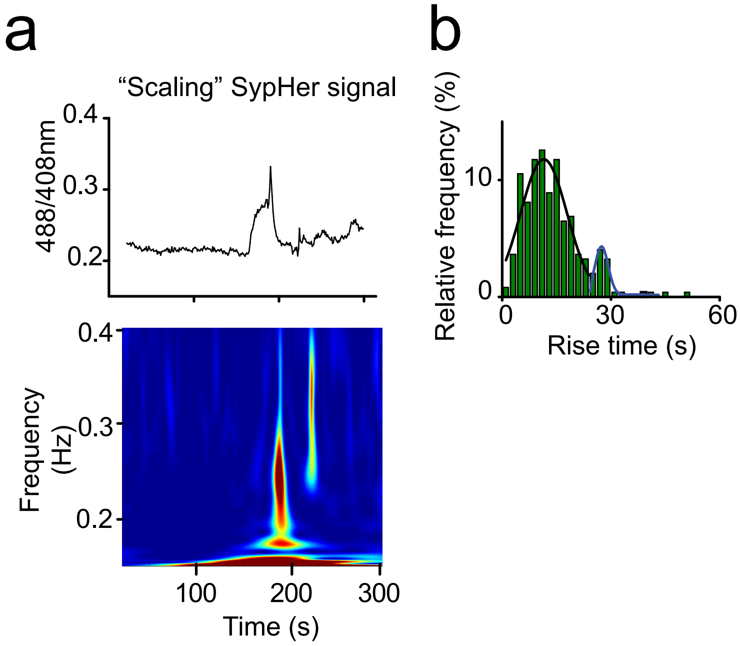

Suppl. Fig.8

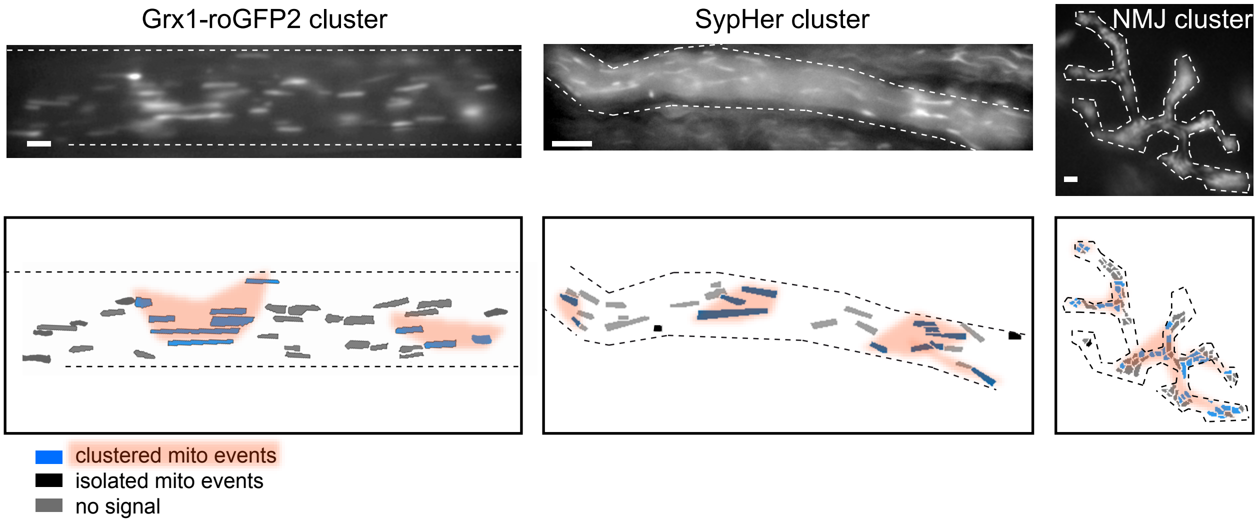

Suppl. Fig.9

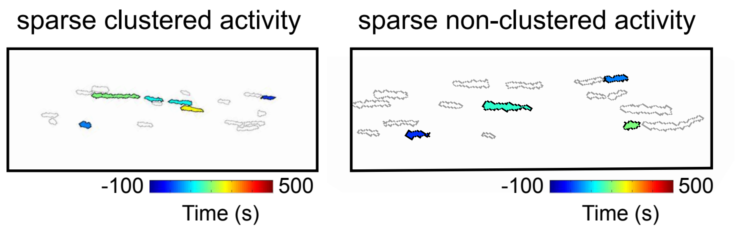

Table 1: Signal distribution

# of signals	0	1	2	>2	Total
Grx1-roGFP2	600 (73%)	150 (18%)	29 (3%)	38 (4%)	817
SypHer	156 (48%)	107 (33%)	34 (5%)	25 (5%)	322
NMJ	140 (54%)	70 (27%)	22 (8%)	25 (9%)	257
MitoQ	155 (82%)	27 (14%)	2 (1%)	3 (1%)	187
MitoQ-ctrl	95 (71%)	33 (24%)	3 (2%)	2 (2%)	133
crush:	23 (28%)	8 (9%)	0 (0%)	50 (61%)	81
total (n)	1169	395	90	143	1797

Table 2: Signal characteristics

mito-Grx1-roGFP2

# of signals	1	2	>2	average
amplitude	0.10 ± 0.01	0.07 ± 0.01	0.05 ± 0.01	0.09 ± 0.01
dT up (s)	47.1 ±1.9	43.6 ± 2.4	46.2 ± 4.01	46.5 ± 1.6
max. slope	0.003 ± 0.001	0.001 ± 0.001	0.001 ± 0.001	0.002 ± 0.001
decay slope	-0.029 ± 0.002	-0.040 ± 0.006	0.051 ± 0.012	-0.031 ± 0.002
down ampl.	0.063 ± 0.005	0.044 ± 0.005	0.030 ± 0.002	0.058 ± 0.003
dT down (s)	96.0 ± 4.6	67.9 ± 7.9	57.0 ± 8.5	88.5 ± 4.0
total (n)	150	29	38	817

mito-SypHer

# of signals	1	2	>2	average
amplitude	0.08 ± 0.01	0.08 ± 0.01	0.07 ± 0.01	0.08 ± 0.01
dT up (s)	15.9 ± 0.9	14.3 ± 0.1	12.1 ± 0.9	14.9 ± 0.6
max. slope	0.008 ± 0.001	0.006 ± 0.001	0.0069 ± 0.0007	0.0077 ± 0.0009
decay slope	-0.1382 ± 0.007	-0.148 ± 0.010	-0.1373 ± 0.0089	-0.140 ± 0.005
down ampl.	0.1011 ± 0.008	0.09 ± 0.01	0.0697 ± 0.0063	0.093 ± 0.006
dT down (s)	29.0 ± 2.5	22.4 ± 2.0	18.9 ± 1.3	26.0 ± 1.7
total (n)	102	32	26	160

Supplementary Figures:

Suppl. Fig. 1: Signal characterization
Example trace of Grx1-roGFP2 oxidation during a single contraction illustrates the determination of up and down slope, signal amplitude and time intervals. For each mitochondrial signal s(t) manual trace characterization determined the start and end points of the corresponding signal rise and decay time-intervals (Δtup, Δtdown). The amplitude (ΔA) is the maximum signal rise above baseline. The corresponding rise (decay) slopes were determined with a linear (exponential) fit of s(t) according to $s(t) = s0 + c \cdot t$ ($s(t) = s0 + b \cdot \exp(-a \cdot t)$).

Suppl. Fig. 2: Various signal forms of oxidizing mitochondria
Example traces of Grx1-roGFP2 mitochondria show a variety of traces that include no signal, single, double or multiple oxidations of single organelles.

Suppl. Fig. 3: Cervical spinal cord, peripheral nerve and NMJ of a wildtype mouse injected with AAV-mito-SypHer Representative image of a horizontal spinal cord section of a wildtype mouse, which was bilaterally injected with AAV-mito-SypHer two weeks prior (a). Magnified images show motorneurons in the ventral horn with long projections that give rise to intercostal axons. Three axons in the intercostal nerve with virally labelled mitochondria (b). Note that there is also fluorescent protein in the cytoplasm of the axons. Image of a virally labelled NMJ (c). Scale bar is 200 µm in (a), 10 µm in close ups and 2 µm in (b) and (c).

Suppl. Fig. 4: Signal characteristics and morphological parameters of mitochondria
Mitochondrial signal rise and decay times (a,b), mitochondrial shape factor (c), area (d), signal amplitude (e) and gap distance are shown (f). ***p<0.001.

Suppl. Fig. 5: Baseline oxidation and pH of contracting and non-contracting mitochondria.
Baseline EGSH oxidation and mitochondrial matrix pH of contracting and non-contraction mitochondria. *p<0.05, **p<0.01.

Suppl. Fig. 6: Signal characteristics of redox and pH signals
Non-, single- and multi-event intensity traces for individual mitochondria as well as their associated absolute squared wavelet transforms (lower panels) are shown for Grx1-roGFP2 (a) and SypHer (b), respectively. While non-events do not exhibit any relevant frequency features, single-event signals are associated with a wavelet transform that is smeared around the inverse signal length (~ 5-10 mHz for Grx1-roGFP2 and ~ 50-100 mHz for SypHer). For multi-event traces, the wavelet transform illustrates the additional frequency of subsequent events (e.g. ~ 8 mHz for Grx1-roGFP2 doublet and ~ 10-20 mHz for Grx1-roGFP2 multi-events, and ~ 10-30 mHz for the SypHer multi-event trace; see also dashed line in wavelet transforms).

Suppl. Fig. 7: Illustration of mitochondrial scaling
Single trace of a representative pH spike exhibiting frequency scaling behavior (a). The absolute squared wavelet transform of the main signal (~ 30 mHz) contains high-frequency content in the buildup to the signal peak at ~180 s (b). Rise time distribution of SypHer signals (c).

Suppl. Fig. 8: Spatial clustering of mitochondrial signals
Representative axons and NMJ are shown with respective spatial clusters of mitochondrial signals (lower panel). Mitochondria that exhibit an event during the recording are depicted in blue when they are part of a spatial cluster, in black when they are not part of a spatial cluster, and in grey when they do not exhibit any event. Red outline around mitochondria indicates the respective cluster. Dashed line shows the axon / NMJ border. Scale bars are 2 µm.

Suppl. Fig. 9: Isochrone analysis
Some axons show only sparse clustered activity or no apparent event clustering. This might be due to mitochondrial signals in the axon being normally distributed with local hotspots and less active regions.
